# Supplementary material for: Dysregulated Metabolic Pathways in Subjects with Obesity and Metabolic Syndrome
Source: Int J Mol Sci. 2022 Aug 29;23(17):9821. doi: 10.3390/ijms23179821 (PMC9456113; doi:10.3390/ijms23179821)
Supplement: Supplementary file 1 [file ijms-23-09821-s001.zip › ijms-1818817-supplementary.pdf]

Dysregulated Metabolic Pathways in Subjects with Obesity and Metabolic Syndrome

Fayaz Ahmad Mir [1,†,*], Ehsan Ullah [2,†,*], Raghvendra Mall [2,3], Ahmad Iskandarani [1], Tareq A. Samra [1], Farhan Cyprian [4], Aijaz Parray [5], Meis Alkasem [1], Ibrahem Abdalhakam [1], Faisal Farooq [2] and Abdul-Badi Abou-Samra [1]

1 Qatar Metabolic Institute, Academic Health System, Hamad Medical Corporation, Doha, Qatar

2 Qatar Computing Research Institute (QCRI), Hamad Bin Khalifa University, Doha, Qatar

3 Department of Immunology, St. Jude Children's Research Hospital, Memphis 38104, USA

4 College of Medicine, QU Health, Qatar University, Doha, Qatar

5 Qatar Neuroscience Institute, Academic Health System, Hamad Medical Corporation, Doha, Qatar

† These authors contributed equally to the work.

* Correspondence: fmir1@hamad.qa (F.A.M.); eullah@hbku.edu.qa (E.U.)

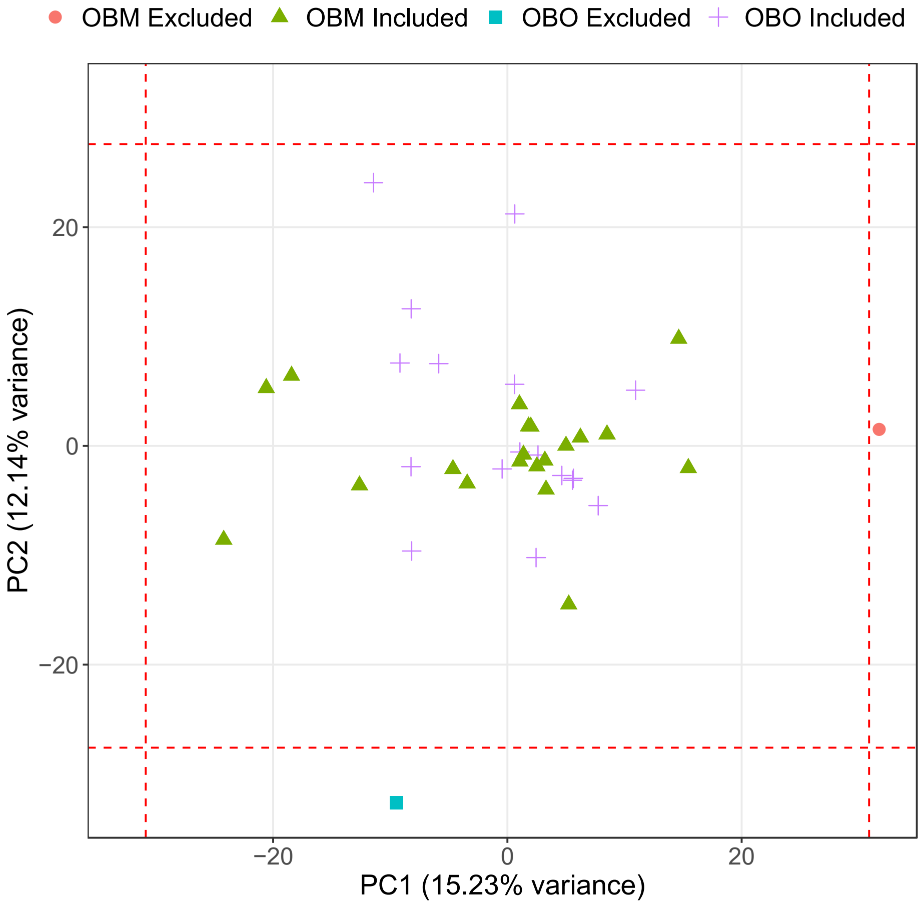

Figure S1. Principal component analysis of samples to identify outliers. Only first two principal components are plotted. Using an outlier threshold level of [μ ± 3SD] on first five principal components, two samples (one OBO and OBM) samples were identified as outliers.

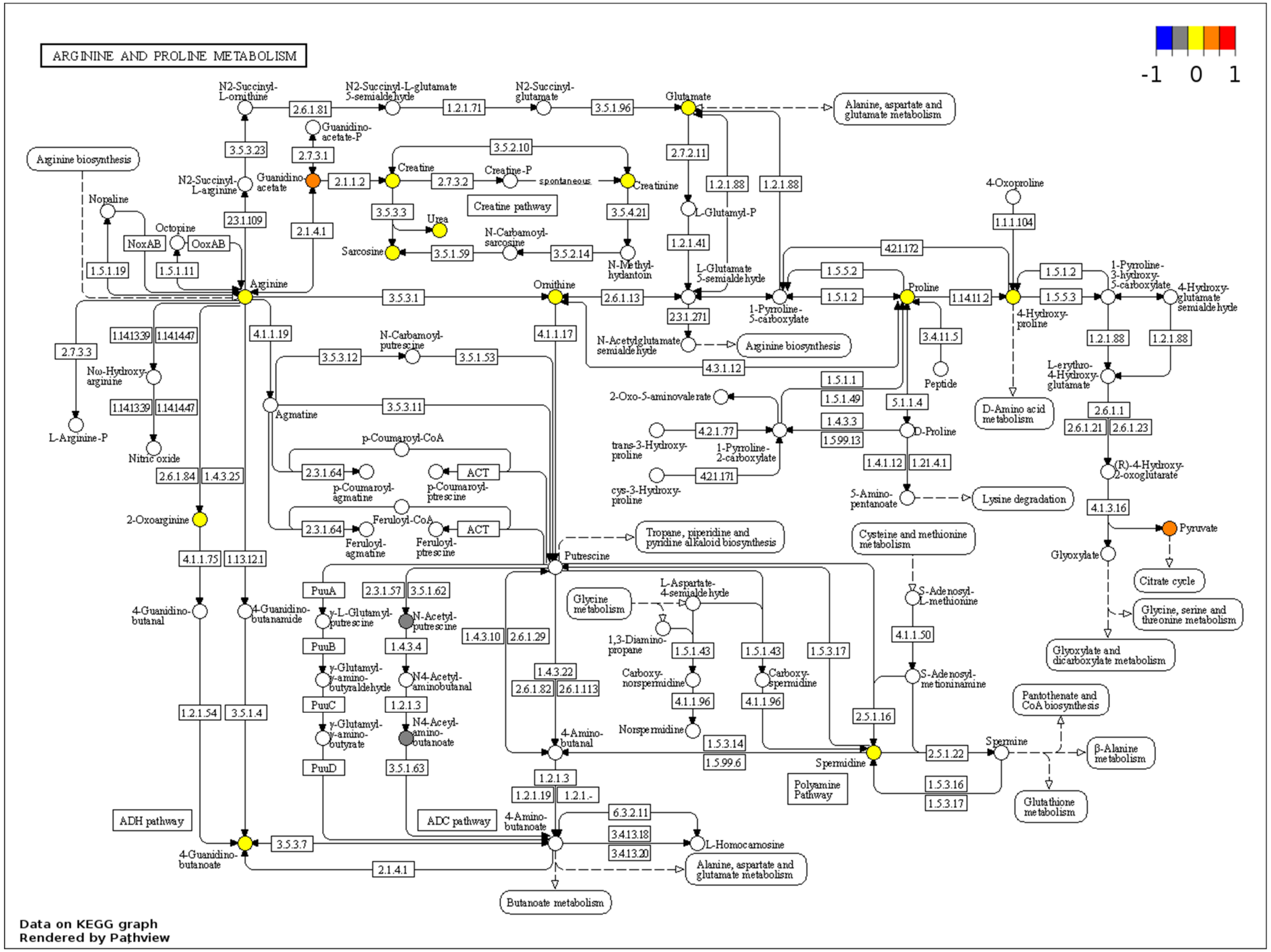

Figure S2. KEGG pathway map of arginine and proline metabolism. Metabolites in blue, yellow, and red have lower, the same, and higher concentration in OBM compared to OBO. Non-colored metabolites were not part of metabolomics profile.
